# Laser Treatment Increases the Antimicrobial Efficacy of Cyanobacterial Extracts against *Staphylococcus* *aureus* (SA) and *Methicillin-resistant* *Staphylococcus aureus* (MRSA)

**DOI:** 10.3390/ijerph192013305

**Published:** 2022-10-15

**Authors:** Haifa M. Al Naim, Nermin El Semary

**Affiliations:** 1 Biological Sciences Department, College of Science, King Faisal University, Al-Ahsa 31982, Saudi Arabia; 2 Botany and Microbiology Department, Faculty of Science, Helwan University, Ain Helwan, Helwan, Cairo 11795, Egypt

**Keywords:** antimicrobial bioassay, aqueous extracts, phycocyanin, *Thermoleptolyngbya* sp., *Leptolyngbya* sp., laser, *Staphylococcusaureus* (SA), *Methicillin-resistantStaphylococcus aureus* (MRSA)

## Abstract

*Staphylococcus aureus* (SA) and *Methicillin-resistant Staphylococcus aureus* (MRSA) are multidrug-resistant bacterial pathogens. A novel approach needs to be followed to combat these pathogens in an ecofriendly manner. Cyanobacterial extracts were previously proven to be affective as antimicrobial agents. To capitalize on this, laser treatments were used to increase their antimicrobial efficacy. Two cyanobacterial strains isolated from Al-Ahsa were identified using molecular methods. Their aqueous extracts were used in the antimicrobial bioassay for these two bacterial pathogens. The first group of aqueous extracts were exposed directly to laser treatment and used in antibacterial bioassay. In parallel, the cyanobacterial biomass of the two isolates was exposed to the laser, then aqueous extracts were prepared. The third group of extracts were not exposed to the laser and were used as a control. Time and distance were the factors tested as they affected the dose of the laser, both individually and in combination. In addition, accessory pigment estimation in extracts before and after laser exposure of extracts was also determined. The two cyanobacterial strains were identified as *Thermoleptolyngbya* sp. and *Leptolyngbya* sp. and the molecular analysis also confirmed the identity of pathogenic bacteria. The untreated cyanobacterial aqueous extracts had little effect against the two bacterial strains. In contrast, the extract directly exposed to the laser was significantly more effective, with an inhibition zone of 22.0 mm in the case of a time of 32 min and distance of 10 cm against *S. aureus*. Accessory pigment composition increased in extracts directly exposed to the laser. This is the first case report on the effect of lasers on enhancing the antimicrobial profile of cyanobacterial extracts against SA and MRSA bacterial pathogens, as well as enhancing accessory pigment content. The laser dose that was most effective was that of 32 min time and 10 cm distance of *Thermoleptolyngbya* sp. extract directly exposed to the laser, which highlights the importance of time for increasing the laser dose and consequently increasing its antimicrobial impact.

## 1. Introduction

Cyanobacteria are oxygenic autotrophs [1]. They are considered a prolific source of bioactive compounds [2]. They are usually produced by specific strains under certain conditions [3]. Secondary metabolism results in the production of biomolecules that have biological effects but are not necessary for growth or reproduction; instead they enable cyanobacteria to survive and thrive. Moreover, they play a protective role against different pathogens [4]. Cyanobacteria contain a wide range of antimicrobial agents that may present alternatives to synthetic antibiotic drugs [5,6]. Most of those agents are released into the environment during growth, while others accumulate in biomass [7,8]. Interestingly, cyanobacterial pigments are reported to have antimicrobial activity [9]. Indeed, phycocyanin pigment proteins present in *Spirulina platensis* showed antibacterial effects [10,11]. It has been reported that fatty acids, flavonoids, and polyphenol compounds were present in cyanobacterial extracts that exhibited antimicrobial activity against Gram-positive and Gram-negative bacteria [12]. Many strains of filamentous cyanobacteria produce several alkaloid compounds that show antimicrobial activity [13]. A class of peptides produced from cyanobacteria showed antimicrobial activity against bacteria [14]. Antibiotics are less effective against Gram-negative bacteria; they possess a multilayered cell wall that impedes antimicrobial agents’ penetration [15].

Cyanobacteria have the ability to produce bioactive compounds that can be potent antimicrobial agents [16]. Cyanobacterial extracts are known for their antibacterial action against a wide array of bacteria. Extracts of *Phormidium* species inhibited the growth of pathogenic strains of *Pseudomonas aeruginosa*, *Staphylococcus aureus*, *Escherichia coli*, and *Streptococcus enteritidis* [17]. Acetone extract of *Oscillatoria agardhii* caused growth inhibition of *Salmonella senftenberg* [18]. Ethanol extracts of *Spirulina platensis* had antimicrobial activity against Gram-negative and Gram-positive bacteria [19]. Ethanol and aqueous extracts of *Anabaena circinalis* showed strong antibacterial activity [20]. Organic extracts of different species of phytoplankton also inhibited the growth of different species of bacteria [21]. Three cyanobacterial strains exhibited significant activity against *Bacillus subtilis* ATCC-11774 and *P. auruginosa* ATCC-15442 bacteria [22]. Moreover, two filamentous genera, *Chroococcus* sp. and *Lyngbya* sp., extracted with several concentrations of three solvents—methanol, ethanol, and diethyl ether—inhibited *S. aureus*, *E. coli*, and *B. subtilus* [23]. Antimicrobial activity of methanolic extracts of *Oscillatoria sancta* and ethanolic extracts of *Lyngbya birgei* showed activity against six species of human pathogenic bacteria [24]. Several cyanobacterial strains were found to possess antibacterial properties against several bacteria [25]. Aqueous extracts of *Microcysties aeruginosa* and *Tychonema bourrellyi* showed antibacterial activity as well [26]. Aqueous and methanolic extracts of different strains of *Nostoc* and *Anabaena* showed allelopathic activity against *B. subtilis*, *S. aureus*, *E. coli*, and *P. auruginosa* [27].

*Methicillin-resistant Staphylococcus aureus* is a Gram-positive bacterium that was discovered in 1961. Through the past few decades, MRSA has evolved and succeeded in resisting antibiotics through both mutation and the gain of exogenous genes that have successfully converted *Staphylococcus aureus* into MRSA, which resists all *β*-lactam antibiotics, resulting in an increase in mortality rates [28,29].

Several reports have shown that cellular functions can be affected by visible light (400–700 nm) irradiation, but the mechanisms must be further investigated [30]. A low-energy laser has been found to modify several biological processes in cell cultures and animal models [31]. Laser irradiation can cause important cellular reproduction and emission of growth factor from cells [32]. Lasers create biological modification, not only through direct radiation effects, but also through thermal effects [33]. In addition, the laser can be used as a mode of therapy. The laser permits surgeons to carry out their work with high precision by targeting a small area, minimizing damage to the surrounding tissue [34]. Laser therapy causes less pain, scarring, and swelling to patients than traditional surgery. Laser therapy is used to shrink and destroy tumors, eliminate kidney stones, repair detached retinas, and treat hair loss [35]. The laser can also seal wounds. They can be used to seal nerve endings to reduce pain after surgical procedures and to seal blood and lymph vessels to reduce swelling and limit the spread of tumor cells. However, there is a paucity of literature on the use of lasers for increasing the antimicrobial bioactivity of entities from algal/cyanobacterial cells.

To investigate this, the antimicrobial potential of some of the cyanobacterial strains and the effect of laser treatment on the antimicrobial activity of their extracts were tested. Furthermore, we investigated the effect of different doses of laser treatments on the antimicrobial activity of both extracts derived from cyanobacterial biomass pre-exposed to lasers and extracts directly exposed to lasers in an attempt to produce highly efficient antimicrobial agents as an ecofriendly biocontrol strategy to combat pathogenic bacteria without harming humans or the environment.

## 2. Materials and Methods

### 2.1. Isolation, Purification, and Morphological Identification of Cyanobacterial Samples

For this study, cyanobacterial samples were isolated from freshwater locations in the Al-Ahsa region, KSA. The samples were collected from sulfur spring water and Hassawi rice field in May 2019. Cyanobacteria samples were isolated by centrifugation using HERMLEZ200A centrifuge (Germany) at 3000 rpm for 30 min. The biomass was harvested and inoculated in BG-11 growth medium. The freshwater samples were cultured in solid and liquid BG-11 culture medium [36,37,38]. Two cyanobacterial cultures were established and examined by light microscopy using OLYMPuS-Cx23 Microscope (Japan) with 40X objective lens and 100X oil immersion lenses. Light microscopy was initially used to identify the general morphology of the isolated cyanobacterial monocultures. *Leptolyngbya* sp. was isolated from spring water, and *Thermoleptolyngbya* sp. was isolated from the Hassawi rice field. To purify monocultures of Cyanobacteria, a method was followed in which one gm of the wide-spectrum antibiotic Augmentin, containing 875 mg amoxicillin and 125 mg clavulanic acid, was used [39,40].

### 2.2. Pathogenic Bacterial Samples Used in Antimicrobial Bioassay

Two species of pathogenic bacteria were isolated: *Staphylococcus aureus* (SA) and *Methicillin-resistant Staphylococcus aureus* (MRSA) [40,41]. Those species were isolated and purified in the facility of the College of Medicine in King Faisal University in the Al-Ahsa region according to Aldayel and El Semary [42]. They were spread on nutrient agar plates (HIMEDIA) and incubated at 30 °C for 24 h. One colony of bacteria was streaked on nutrient agar plate and incubated at 30 °C for 24 h. Pure colonies of bacterial samples were inoculated on 70% broth media (HIMEDIA) and 30% glycerol (*v/v*) and incubated at 30 °C for 24 h. They were kept in a fridge at 4 °C for future experiments. All bacterial isolates were subjected to Gram staining according to Claus [43].

### 2.3. Initial Screening of Cyanobacterial Extracts and Antimicrobial Bioassays against Pathogenic Bacteria

Two cyanobacterial species were isolated from Eastern region in the Al-Ahsa region, KSA, from sulfur spring water and Hassawi rice field. Individual antimicrobial tests against different pathogenic bacteria with two replicates were performed. One-month-old cyanobacterial cultures were centrifuged at 3000 rpm for 30 min. The biomass was resuspended in water and centrifuged. About 5 g of freshwater cyanobacterial biomass/1 mL distilled water (*w/v*) were used for each sample. The antibacterial bioassay was initially carried out on their aqueous extracts with no laser exposure. The extracts were prepared by homogenizing 1 mg fresh weight of cyanobacterial biomass in 1 mL sterilized distilled water (*w/v*). One colony of bacteria was inoculated in 10 mL of broth media and incubated at 30 °C overnight. Then, they were mixed with 100 mL of sterilized liquid nutrient agar. The disc diffusion method was used to assess the antimicrobial activity of cyanobacterial biomass and cyanobacterial extracts. Sterilized diffusion discs 6 mm in diameter were prepared with the susceptibility method modified by Kirby Bauer [44]. Diffusion discs were saturated with 20 µL of cyanobacterial aqueous extracts and placed in the agar plates that had been inoculated with bacterial strains according to El Semary [45] and Heidari [46]. The agar plates were then incubated at 37 °C overnight.

### 2.4. Molecular Identification of Cyanobacterial Strains

Two filamentous cyanobacterial strains were identified using a molecular method. Universal primers specific for the partial 23S_rDNA genetic marker were used [47]. The following steps were applied for the molecular characterization of the strains. Polymerase chain reaction (PCR) was used to amplify the genetic locus using this primer pair.

#### 2.4.1. DNA Extract of Cyanobacterial Strains

DNA was extracted using a modified method based on [48] in the following protocol:

A freshly-centrifuged pellet of cyanobacterial biomass of weight 20 mg was ground with a pestle in a 1.5 mL tube together with 500 µL of Dellaporta buffer (100 mM Tris pH = 8. 50 mM EDTA, 500 mM NaCl, 10 mM beta mercaptoethanol. This was followed by the addition of 33 µL of 20% sodium dodecyl sulfate (SDS) (*w/v*), and the mixture was vortexed and incubated for 10 min at 65 °C. Then, 160 µL of 5 M potassium acetate (Sigma Chemicals) was added and the mixture was vortexed again. The mixture was spun for 10 min at 10,000 rpm in a microcentrifuge tube and 450 µL of supernatant was transferred to a new tube.

Following this, 450 µL was added to a mixture of phenol, chloroform, and isoamyl-alcohol prepared in a ratio of 25:24:1. The whole mixture was vortexed for 5 min and then spun for 5 min at 10,000 rpm. Four hundred microliters of the upper phase was transferred to a clean microcentrifuge tube and 0.5 volumes of isopropanol was added, vortexed, and spun for 10 min at 14,000 rpm. The supernatant was discarded and the total nucleic acid precipitated in the bottom of the tube. The pellet was washed with 70% ethanol and spun for 5 min at 10,000 rpm. The pellet was resuspended in 100 µL of double-distilled water (dd H_2_O).

#### 2.4.2. PCR Amplification of Cyanobacterial Genomic DNA

The universal primer pair, p23SrV_f1 (5′ GGA CAG AAAGAC CCT ATG AA 3′) and p23SrV_r1 (5′ TCA GCCTGT TAT CCC TAG AG 3′), was used [49].

The main PCR steps were programmed as follows:

1. Initial denaturation at 94 °C for 2 min.
2. Amplification cycles (35×). Each cycle consists of:
  - Denaturation at 94 °C for 20 s;
  - Annealing at 55 °C for 30 s;
  - Extension at 72 °C for 10 s;
3. Final extension step at 72 °C for 10 min [47].
4. The amplicons were purified and sequenced (Macrogen, South Korea), and the sequences were checked in Genbank for similarity check (Blastn). The sequences were deposited in GenBank to await accession number designation.

### 2.5. Laser Treatments Using Different Exposure Time Intervals and Distances

The cyanobacterial cultures used in experiments were one month old. To obtain the highest antimicrobial action, we applied laser radiation treatments to two sets: one set in which laser was applied directly to the cyanobacterial extracts, and the other set involving exposing the cyanobacterial biomass to laser treatment followed by aqueous extraction. The diode laser used had a wavelength of 635 nm with a probe tip 3 mm in diameter and power density of <1 mW in continuous irradiation mode. Aqueous extracts of the two cyanobacteria were tested in antimicrobial bioassay. Both cyanobacterial biomass and extracts were exposed to laser radiation directly for periods of 4, 16, and 32 min at distances of 5 cm and 10 cm from the laser source. The final sample was not subjected to laser radiation and was used as a control. Before conducting the antibacterial bioassay, the aqueous extracts were incubated at 37 °C for 72 h. This was performed for laser-treated extracts as well as extracts derived from laser-treated cyanobacterial biomass. Aqueous extracts were incubated at 37 °C for 72 h after laser treatment.

### 2.6. Measurement the Phycobiliprotein Pigments Quantity of Cyanobacterial Extracts Spectrophotometrically before and after Exposing to Laser

The pigment composition of the extracts of two species of cyanobacteria, *Thermoleptolyngbya* sp. and *Leptolyngbya* sp., were measured by spectrophotometer to compare the quantity of three phycobiliprotein accessory pigments (Phycocyanin, allophycocyanin, phycoerythrin) before and after laser exposure. The concentration of phycobiliprotein pigments were calculated according to the follow equations [50,51,52]:

Phycocyanin (mg/mL) = [A615 nm − 0.474 (A652 nm)]/5.34(1)

Allophycocyanin (mg/mL) = [A652 nm − 0.208 (A615 nm)]/5.09(2)

Phycoerythrin (mg/mL) = [A562 nm − 2.41 (phycocyanin)] − 0.849 (allophycocyanin)/9.62(3)

### 2.7. Statistical Analysis

The statistical design was split-plot based on extracts from pre-exposed biomass or extract exposed directly to laser, which represented the main two plots, while distance was presented as subplots and exposure time sub-subplots. Data were statistically analyzed with the ANOVA of statistics 6 software from the StatSoft company (Statsoft, 2001) and the significance of differences among means was calculated with the least significant difference test (LSD) at *p* < 0.05.

## 3. Results

### 3.1. Molecular Identification of Cyanobacterial Isolate Strains

**Figure 1:**
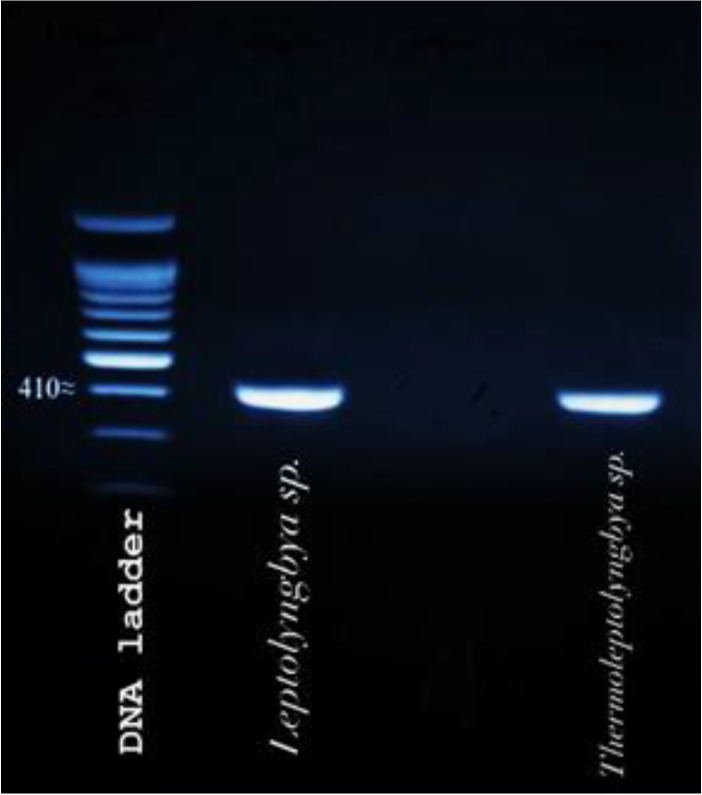
PCR products of the partial 23S rDNA amplification.

The partial 23S rDNA genetic locus of those cyanobacterial strains whose extracts showed significant antimicrobial activity was amplified using universal algal primers developed by Sherwood and Presting [47]. The size of the PCR product was nearly 410 bp according to the DNA ladder (Figure 1). The identities of the two cyanobacterial isolates and their best matches were retrieved via BLASTn search of species and their molecular characterizations was based on close similarity to sequences deposited in Genbank.

### 3.2. Phylogenetic Inference

**Figure 2:**
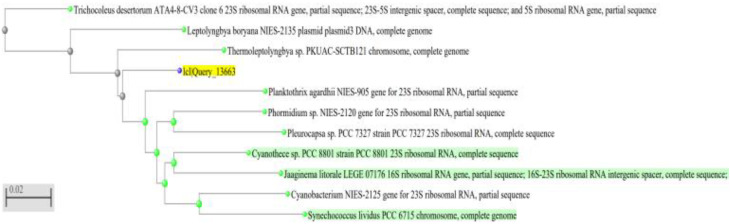
A neighbor-joining phylogenetic tree of *Thermoleptolyngbya* sp. The sequence of the organism under study is highlighted in yellow. Green highlights reference database sequences.

**Figure 3:**
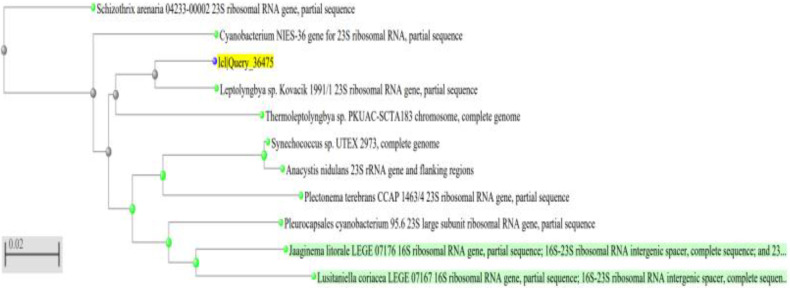
A neighbor-joining phylogenetic tree of *Leptolyngbya* sp. The sequence of the organism under study is highlighted in yellow. Green highlights reference database sequences.

**Figure 4:**
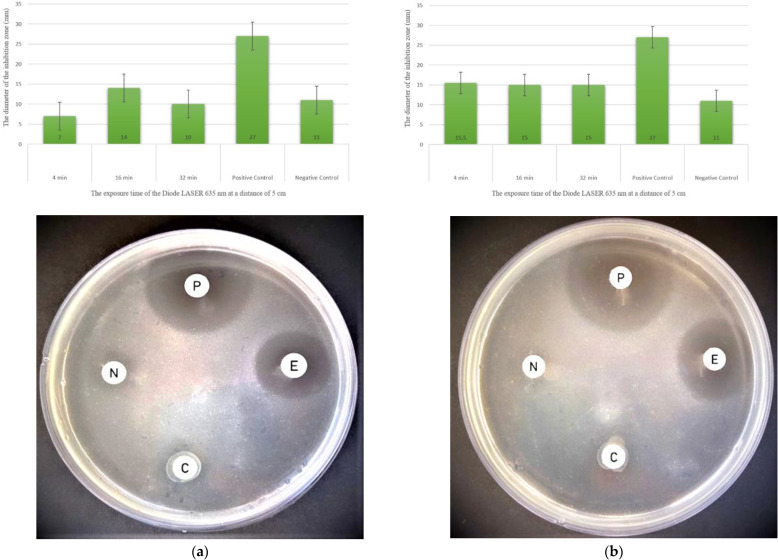
Effect of laser treatment by different exposure times at a distance of 5 cm on *Thermoleptolyngbya* sp. extracts derived from cyanobacterial biomass pre-exposed to laser and extracts directly exposed to laser against *Staphylococcus aureus*. (P)—positive control; (N)—negative control; (C)—an extract derived from cyanobacterial biomass pre-exposed to laser; (E)—an extract directly exposed to laser. (**a**) An agar plate showing the highest inhibition zone of extracts derived from biomass pre-exposed to laser for 16 min. (**b**) An agar plate showing highest inhibition zone of extracts directly exposed to laser for 4 min.

A neighbor-joining phylogenetic tree for all cyanobacterial strains was constructed using the 23S rDNA sequences most similar to the sequences retrieved (Figure 2, Figure 3 and Figure 4). Comparative sequence analysis of the 23S_rRNA gene revealed that:

1. The filamentous unbranched nonheterocystous strain that was isolated from the thermal sulfur water spring belonged to *Thermoleptolyngbya* sp., which exhibited 92.92% sequence homology.
2. The filamentous unbranched nonheterocystous strain that was isolated from the Hassawi rice field belonged to *Leptolyngbya* sp., which exhibited 94.58% sequence homology.

The neighbor-joining phylogenetic tree showed a close relationship of the two filamentous isolates to genera *Thermoleptoyngbya* sp. and *Leptoyngbya* sp., respectively, which are unbranched filamentous cyanobacterial strains. It is worth noting that indeed the filamentous isolate *Thermoleptolyngbya* sp. was actually isolated from the hot sulfur spring in Al-Ahsa, KSA, which reinforces its identity as a thermal isolate.

### 3.3. Molecular Identification of Bacteria

Using the 16S rDNA sequences for pathogenic bacteria, the genetic sequences of the PCR products from bacteria were retrieved after sequencing. The PCR products were nearly 1500 bp. The identities of the two bacterial isolates and their best matches were retrieved via BLASTn search.

### 3.4. Antimicrobial Activity of Cyanobacterial Extracts against Pathogenic Bacteria

Aqueous extracts of *Thermoleptolyngbya* sp. and *Leptolyngbya* sp. derived from cyanobacterial biomass pre-exposed to lasers and the aqueous extracts directly exposed to laser irradiation had significantly enhanced antimicrobial effects against *Staphylococcus aureus* and *Methicillin-resistant Staphylococcus aureus*. Direct laser treatment of extracts resulted in a much higher inhibitory effect than that of the untreated cyanobacterial extract used as a negative control (Table 1 and Table 2). The extract derived from cyanobacterial biomass pre-exposed to the laser of *Thermoleptolyngbya* sp. and its aqueous extract directly exposed to the laser showed broad-spectrum antimicrobial action against all pathogenic bacteria species tested: *S. aureus* and *Methicillin-resistant S. aureus* (Figure 4).

Figure 4 shows the measurement of the antimicrobial effect of *Thermoleptolyngbya* sp. against *Staphylococcus aureus*, where the inhibition zone for the negative control was 11 mm. The antimicrobial effect of extracts treated at a distance of 5 cm for 4 min, 16 min, and 32 min was recorded as follows: 7 mm, 14 mm, and 10 mm, respectively, for *Thermoleptolyngbya* sp. extract derived from cyanobacterial biomass pre-exposed to the laser. Under the same parameters, *Thermoleptolyngbya* sp. extract directly exposed to the laser exhibited a much greater antimicrobial effect, with inhibition zones recorded at 15.5 mm, 15 mm, and 15 mm, respectively.

The inhibition zone for the negative control was 9 mm. At a treatment distance of 5 cm for 4 min, the diameter of the inhibition zone was 6.5 mm for *Thermoleptolyngbya* sp. extract derived from cyanobacterial biomass pre-exposed to the laser, less than that of the negative control. When the dose time was increased to 16 min, the inhibition zone increased to 10 mm. At exposure times of 32 min, the largest inhibition zones were recorded but were not statistically significant at 12 mm for *Thermoleptolyngbya* sp. extract derived from cyanobacterial biomass pre-exposed to laser. In contrast, the results of *Thermoleptolyngbya* sp. extracts directly exposed to laser for all time intervals were statistically significant. At a treatment distance of 10 cm, the inhibition zone diameters of *Thermoleptolyngbya* sp. extracts derived from cyanobacterial biomass pre-exposed to the laser were insignificant for all exposure times. In contrast, *Thermoleptolyngbya* sp. extracts directly exposed to the laser displayed a steadily increasing inhibition zone, with treatment times of 12.5 mm, 14.5, and 16 mm. The results were statistically significant. The best results derived from all cyanobacterial extracts were found in extracts directly exposed to different doses of laser. The largest statistically significant inhibition zones highlighted (Table 1 and Table 2) of these extracts against all tested pathogenic bacteria are as follows:

1. *Thermoleptolyngbya* sp. extracts directly exposed to laser against *Staphylococcus aureus*, with an inhibition zone of 22 mm at 10 cm and 32 min.
2. *Thermoleptolyngbya* sp. extracts directly exposed to the laser *against Methicillin-resistant Staphylococcus aureus*, with an inhibition zone of 19 mm at 5 cm and 16 min.

### 3.5. Measurement the Phycobiliprotein Pigments Quantification of Cyanobacteria by Spectrophotometric

The concentration of three phycobiliprotein pigments (phycocyanin, allophycocyanin, phycoerythrin) of *Thermoleptolyngbya* sp. and *Leptolyngbya* sp. were measured by spectrophotometer before exposure to the laser and after exposure to the laser at 10 cm for 32 min of *Thermoleptolyngbya* sp., as well as at 5 cm for 4 min of *Leptolyngbya* sp. These results were significantly different (*p* < 0.05) from the laser-untreated samples (Table 3):

## 4. Discussion

The aim of this study was to investigate the antimicrobial potential of some of the cyanobacterial strains isolated from the Al-Ahsa region and the effect of laser treatment on the antimicrobial activity of their extracts. The strains’ antimicrobial potential was tested and their identity was confirmed using molecular methods. Furthermore, we investigated, for the first time, the effect of different doses of laser treatment on the antimicrobial activity of both extracts derived from cyanobacterial biomass pre-exposed to a laser and extracts directly exposed to a laser in an attempt to produce highly efficient antimicrobial agents as an ecofriendly biocontrol strategy to combat pathogenic bacteria without harming human or environmental health.

Cyanobacteria are oxygenic autotrophs which produce a wide array of bioactive compounds, including antimicrobial agents [1,16]. The Kingdom of Saudi Arabia represents a rich resource of microflora of exceptional metabolism, especially cyanobacteria from Al-Ahsa oasis, where there are unique sulfur springs and the Hassawi rice fields. Therefore, they represent a possible source of powerful antimicrobial compounds. To explore this hypothesis, we tested aqueous extracts from local cyanobacterial strains isolated from samples taken in different locations in Al-Ahsa against *Staphylococcus aureus* (SA) and *Methicillin-resistant Staphylococcus aureus* (MRSA). Aqueous extracts for two cyanobacterial strains, *Thermoleptolyngbya* sp., and *Leptolyngbya* sp., were found to possess potent antibacterial bioactivity. *Thermoleptolyngbya* sp. was isolated from sulfur spring water and *Leptolyngbya* sp. was isolated from the Hassawi rice fields. To investigate the effect of different red visible-light diode 635 nm laser doses on the antimicrobial activity of the cyanobacterial extracts, both the extracts derived from cyanobacterial biomass pre-exposed to the laser and those directly exposed to the laser were tested against two pathogenic bacteria: *S. aureus* (SA) and *Methicillin-resistant S. aureus* (MRSA), which have a wide host range. The aqueous extracts of untreated cyanobacterial biomass showed limited effects against the two species of pathogenic bacteria. We chose to take the aqueous extract instead of organic extracts as aqueous extracts are easily prepared, ecofriendly, and cost-effective [53,54]. Water is a universal solvent, widely available and safer to use without causing harm to organisms, health, or the environment. Indeed, Martel et al. reported that methanolic extracts are more toxic than aqueous extracts [55]. To concentrate bioactive compounds in aqueous extracts as well as to enhance antimicrobial activity, aqueous extracts were incubated at 37 °C for 72 h after laser treatment. This step increased the inhibition zone diameter considerably. Incubation allowed excess water to evaporate, thereby increasing the concentration of the bioactive compounds, as Monserrat et al. described in concentrating aqueous extracts of *Anabaena spiroides* at 40 °C [56]. The cyanobacterial aqueous extracts not exposed to laser were used as controls to show the significance of difference in antimicrobial activity between laser-treated and untreated extracts. Indeed, laser treatments significantly increased the antimicrobial activity, with the highest inhibition zone diameter observed in *Thermoleptolyngbya* sp. against *S. aureus;* this only emphasizes the significance of laser treatment on the antibacterial activity of extracts. Cyanobacteria can respond to different environmental stresses such as radiation by producing unique metabolites or increasing the production of certain metabolites such as pigments that can have exceptional antimicrobial effects. Manipulating the stress conditions by exposing cyanobacteria to irradiation increased the production of bioactive compounds, thus increasing their antimicrobial activity. Exposing cyanobacteria and their aqueous extracts to irradiation significantly enhanced their antimicrobial activity. This agrees with Aldayel and El Semary et al. (2020), who reported the occurrence of a considerable enhancement in antimicrobial activity after exposing cyanobacterial strains to ultraviolet (UV) and gamma radiation in order to enhance their antimicrobial activity [57]. However, the UV-B irradiation causes a decrease in photosynthesis by both a direct effect on the photosystem and an indirect effect by decreasing the pigment content, thereby causing a lower biomass of cyanobacteria [58]. In addition, UV irradiation has mutagenic and carcinogenic effects that cause cellular damage [59]. On the other hand, gamma radiation is ionizing radiation that can cause DNA mutation and change in the physiological and morphological properties of organisms [60]. Because this form of irradiation can cause mutation and DNA damage as well as changing the natural properties of cyanobacteria, the possibility of introducing mutant organisms into the environment poses a high risk of unpredictable and potentially hazardous side effects. Thus, it is better to avoid ionizing radiation and to adopt the novel approach of applying non-ionizing visible radiation, such as the laser radiation we used in this study to avoid the hazards that result from ionizing radiation. Hence, we used the visible red-light radiation of a 635 nm laser beam to ensure that the cyanobacteria were not subjected to genetic change. Thus, the risks are minimized and the antimicrobial effectiveness is maximized.

We also used two different modes of application of the laser: first by exposing the cyanobacterial biomass, then preparing the extract or exposing the extract directly to laser treatment in order to compare the antimicrobial action. Generally, the results of this experiment indicated that the radiation increased the antimicrobial activity of cyanobacteria both in the cyanobacterial biomass that was pre-exposed to the laser and also in the extracts exposed directly to the laser, but the latter proved to be more active against pathogenic bacteria. The improvement of antimicrobial activity by radiation is mostly attributed to the increase in the synthesis of antimicrobial compounds in cyanobacteria. The effect of radiation depends on the type of radiation to which cyanobacteria are subjected to, as well as the doses and solvent used. This is similar to Fatima et al., who found that they attributed the effect of different doses of UV-B radiation on the *Synechococcus* spp. PCC7942 against pathogenic bacteria to the inhibitory impact of carotenoids and phycocyanin [61]. In contrast, Doman et al. showed that UV-B radiation caused an increase in the biomass, chlorophyll A, protein, and carbohydrates of *Spirulina platensis,* as well as induced an increase in the biosynthesis of carotenoids, scytonemin, phycoerythrin, and allophycocyanin and phycocyanin degradation [62]. Both light intensity and quality can impact photosynthetic pigments in cyanobacteria. The control of light conditions by synthetic light can be a sufficient factor to affect cyanobacteria pigment production. This is in agreement with Kim et al., who reported that light-emitting diodes (LED) are suitable artificial light and economic energy sources to increase the cyanobacterial pigment content [63]. A light-emitting diode can provide the red light [64] that we used in our experiment. Monochromatic light can affect photosynthesis and pigment production [65]. Indeed, the quality of light can influence the photosynthetic pigments’ quantity. For example, Sivasankari et al. proved that cyanobacteria that grow in red light contain a higher concentration of phycocyanin than cyanobacteria grown in white light without cell dispersion. In our case, it is most likely that exposure of cyanobacteria to a red-light laser has resulted in an increase in the phycocyanin content. This particular pigment has antimicrobial activity against pathogenic bacteria [66]. This may account for the increase in the antibiosis after exposure to the red-light laser, as well as accounting for the increase in accessory pigments that have antimicrobial potential as a result of laser treatments. In the present study, a red visible-light diode set at 635 nm provided a laser at an effective wavelength, which induced enhancement in antimicrobial activity. It was also efficient in terms of safety, efficacy, and reproducibility. This is in agreement with Wiench et al., who found that the diode 635 nm laser does not cause cytotoxicity, and is therefore safe and easy to use [67]. Furthermore, different doses of diode 635 nm laser treatment were investigated by changing the distances and exposure times to determine the most potent doses for highest antimicrobial bioactivity. Indeed, a previous study showed that the penetration and absorption of laser radiation in biological cells depend on several factors, including the distance between the laser source and the cell, the area of the cell exposed to the laser radiation, laser wavelength, treatment time, dose, and power density [68]. To account for these factors, the cyanobacterial biomass and extracts were exposed to different doses of the diode 635 nm laser by varying the distance (5 cm and 10 cm) and exposure time (4 min, 16 min, 32 min) of treatment. The experimental results showed that the highest inhibition zone diameter was obtained in *Thermoleptolyngbya* sp. extracts compared with extracts of *Leptolyngbya* sp. The *Thermoleptolyngbya* sp. was isolated from sulfur spring water, where the temperatures were high-degree. Interestingly, Tang et al. has proven that the *Thermoleptolyngbya* sp. abundant in thermal environments can adapt to hot spring niches [69]. The higher temperatures engender a physiological and genetic change in *Thermoleptolyngbya* sp. to resist the destructive effect of high temperatures. They also possess unique metabolisms and exceptional bioactive compounds. Yoon et al. proved that more than 100 sequences of the cyanobacteria inhabiting hot springs that matched *Leptolyngbya* sp. possessed powerful biotechnological potential [70,71]. However, because *Thermoleptolyngbya* sp. has thermal adaptability, the new genus of *Thermoleptolyngbya* sp. was described and proposed the novel classification within the family Leptolyngbyaceae, order Synechococcales [72,73]. Cyanobacteria that belong to the *Thermoleptolyngbya* genus seem to be thermotolerant rather than thermophilic and carry the ability to fix nitrogen [70,72]. Therefore, extracts from this exceptional multitasking cyanobacterium are most likely unique in their metabolism, and it is not surprising that they were effective against those bacterial pathogens. The research presents a novel study of enhancement of the antimicrobial effect of cyanobacterial extracts using non-ionizing visible-light radiation. The study also opens the door for future studies that can reveal more on the role of pigments in responding to radiation and how to exploit this further in antibiosis.

## 5. Conclusions

The exposure of cyanobacterial extracts to varying laser doses significantly increased the antimicrobial activity of different local cyanobacterial strains. The use of a red-light laser caused an increase in the production of accessory pigments of antimicrobial activity, hence increasing their antimicrobial efficacy. This approach is useful and safe and can be applied over a wide range to use those extracts as biocontrol agents that are ecofriendly, cost-effective, and achieve both environmental sustainability and food security.

## Figures and Tables

**Table 1:** Effect of interaction between distance and time intervals as functions of laser dose treatment on the antimicrobial effect of cyanobacterial extracts derived from cyanobacterial biomass pre-exposed to laser and cyanobacterial extracts directly exposed to laser against *Staphylococcus aureus.* The highest statistically significant result (*p* < 0.01) is indicated in green; statistically significant results are indicated in pink (*p* < 0.05); highest but statistically nonsignificant results are indicated in yellow.

Cyanobacterial Strain	Distance	Exposure Time	Inhibition Zone of *Staphylococcus aureus*	
			Extracts from Biomass Pre-Exposed to Laser	Extracts Directly Exposed to Laser
*Thermoleptolyngbya* sp.	5 cm	4 min	7.0 ± 0.0	15.5 ± 7.7
		16 min	14.0 ± 1.4	15.0 ± 0.0
		32 min	10.0 ± 1.4	15.0 ± 7.0
	10 cm	4 min	12.0 ± 1.4	20.0 ± 4.2
		16 min	8.5.0 ± 0.7	19.0 ± 4.2
		32 min	14.5 ± 3.5	22.0 ± 4.2
**Control, no laser treatment**			11 ± 0.0	11.0 ± 0.0
*Leptolyngbya* sp.	5 cm	4 min	8.0 ± 0.0	21.5 ± 3.5
		16 min	12.5 ± 3.5	11.5 ± 2.1
		32 min	8.5 ± 0.7	9.0 ± 0.0
	10 cm	4 min	8.0 ± 1.4	11.0 ± 2.8
		16 min	15.0 ± 2.8	10.0 ± 4.2
		32 min	9.5 ± 0.7	14.0 ± 1.4
**Control, no laser treatment**			9.5 ± 0.0	9.5 ± 0.0

**Table 2:** Effect of interaction between distance and time intervals as functions of laser dose treatment on the antimicrobial effect of cyanobacterial extracts derived from cyanobacterial biomass pre-exposed to laser and cyanobacterial extracts directly exposed to laser against *Methicillin-resistant Staphylococcus aureus*. The highest statistically significant result is indicated in green (*p* < 0.01); statistically significant results are indicated in pink (*p* < 0.05); highest but statistically nonsignificant results are indicated in yellow.

Cyanobacterial Strain	Distance	Exposure Time	Inhibition zone of MRSA-*Staphylococcus aureus*	
			Extracts from Biomass Pre-Exposed to Laser	Extracts Directly Exposed to Laser
*Thermoleptolyngbya* sp.	5 cm	4 min	6.5 ± 0.7	13.5 ± 3.5
		16 min	10.0 ± 1.4	19.0 ± 1.4
		32 min	12.0 ± 4.9	13.5 ± 2.1
	10 cm	4 min	8.5 ± 2.1	13.5 ± 3.5
		16 min	9.0 ± 0.0	14.5 ± 4.9
		32 min	9.5 ± 0.7	16.0 ± 1.4
**Control, no Laser treatment**	-	-	9.0 ± 0.0	9.0 ± 0.0
*Leptolyngbya* sp.	5 cm	4 min	8.5 ± 0.7	13.0 ± 0.0
		16 min	8.5 ± 0.7	8.5 ± 0.7
		32 min	10.5 ± 2.1	11.0 ± 2.8
	10 cm	4 min	11.0 ± 2.3	10.0 ± 2.1
		16 min	8.5 ± 2.1	11.0 ± 2.8
		32 min	9.0 ± 0.0	9.0 ± 0.0
**Control, no Laser treatment**			6.5 ± 0.0	6.5 ± 0.0

**Table 3:** The phycobiliprotein pigments concentration of extracts before and after to laser exposure of *Thermoleptolyngbya* sp. and *Leptolyngbya* sp.

Species	Distance	Time	Phycocyanin (mg/mL)	Allophycocyanin (mg/mL)	Phycoerythrin (mg/mL)
*Thermoleptolyngbya* sp.	0 (Control, no laser treatment)	0 (Control, no laser treatment)	0.0624704	0.0380794	0.205086
	10	32	0.824993	0.118537	0.690715
*Leptolyngbya* sp.	0 (Control, no laser treatment)	0 (Control, no laser treatment)	0.0244599	0.0329147	0.206482
	5	4	0.106869	0.0971033	0.527876

## References

[1] Singh S.P., Häder D.-P., Sinha R.P. (2010). Cyanobacteria and ultraviolet radiation (UVR) stress: Mitigation strategies. Ageing Res. Rev..

[2] Nunnery J.K., Mevers E., Gerwick W.H. (2010). Biologically active secondary metabolites from marine Cyanobacteria. Curr. Opin. Biotechnol..

[3] Martínez-Francés E., Escudero-Oñate C. (2018). Cyanobacteria and microalgae in the production of valuable bioactive compounds. Microalgal Biotechnol..

[4] Mandal S., Rath J. (2015). Secondary metabolites of Cyanobacteria and drug development. Extremophilic Cyanobacteria for Novel Drug Development.

[5] Fernandes P. (2006). Antibacterial discovery and development—The failure of success. Nat. Biotechnol..

[6] Singh S., Kate B.N., Banerjee U.C. (2005). Bioactive compounds from Cyanobacteria and microalgae: An overview. Crit. Rev. Biotechnol..

[7] Jaki B., Orjala J., Heilmann J., Linden A., Vogler B., Sticher O. (2000). Novel extracellular diterpenoids with biological activity from the cyanobacterium Nostoc commune. J. Nat. Prod..

[8] Jaki B., Zerbe O., Heilmann J., Sticher O. (2001). Two novel cyclic peptides with antifungal activity from the cyanobacterium Tolypothrix byssoidea (eawag 195). J. Nat. Prod..

[9] Goud M.J.P., Seshikala D., Charya M.A.S. (2007). Antibacterial activity and biomolecular composition of certain fresh water microalgae collected from river godavari (India). Int. J. Algae.

[10] Muthulakshmi M., Saranya A., Sudha M., Selvakumar G. (2012). Extraction, partial purification, and antibacterial activity of phycocyanin from Spirulina isolated from fresh water body against various human pathogens. J. Algal Biomass Util..

[11] Santoyo S., Herrero M., Senorans F.J., Cifuentes A., Ibáñez E., Jaime L. (2006). Functional characterization of pressurized liquid extracts of Spirulina platensis. Eur. Food Res. Technol..

[12] Abdo S.M., Hetta M.H., Samhan F.A., El-Din R.A.S., Ali G.H. (2012). Phytochemical and antibacterial study of five freshwater algal species. Asian J. Plant Sci..

[13] Kim H., Lantvit D., Hwang C.H., Kroll D.J., Swanson S.M., Franzblau S.G., Orjala J. (2012). Indole alkaloids from two cultured Cyanobacteria, Westiellopsis sp. and Fischerella muscicola. Bioorganic Med. Chem..

[14] Montaser R., Paul V.J., Luesch H. (2011). Pitipeptolides c–f, antimycobacterial cyclodepsipeptides from the marine cyanobacterium Lyngbya majuscula from guam. Phytochemistry.

[15] Amaro H.M., Guedes A.C., Malcata F.X. (2011). Antimicrobial activities of microalgae: An invited review. Sci. Against Microb. Pathog. Commun. Curr. Res. Technol. Adv..

[16] Alsenani F., Tupally K.R., Chua E.T., Eltanahy E., Alsufyani H., Parekh H.S., Schenk P.M. (2020). Evaluation of microalgae and Cyanobacteria as potential sources of antimicrobial compounds. Saudi Pharm. J..

[17] Thummajitsakul S., Silprasit K., Sittipraneed S. (2012). Antibacterial activity of crude extracts of Cyanobacteria *Phormidium* and *Microcoleus* species. Afr. J. Microbiol. Res..

[18] El-Aty A.M.A., Mohamed A.A., Samhan F.A. (2014). In vitro antioxidant and antibacterial activities of two fresh water cyanobacterial species, *Oscillatoria agardhii* and *Anabaena sphaerica*. J. Appl. Pharm. Sci..

[19] Ozdemir G., Karabay N.U., Dalay M.C., Pazarbasi B. (2004). Antibacterial activity of volatile component and various extracts of *Spirulina Platensis*. Phytother. Res. Int. J. Devoted Pharmacol. Toxicol. Eval. Nat. Prod. Deriv..

[20] Shaieb F.A., Issa A.A., Meragaa A. (2014). Antimicrobial activity of crude extracts of Cyanobacteria *Nostoc commune* and *Spirulina platensis*. Arch. Biomed. Sci..

[21] del Pilar Sánchez-Saavedra M., Licea-Navarro A., Bernáldez-Sarabia J. (2010). Evaluation of the antibacterial activity of different species of phytoplankton. Rev. Biol. Mar. Oceanogr..

[22] Malathi T., Babu M.R., Mounika T., Snehalatha D., Rao B.D. (2014). Screening of cyanobacterial strains for antibacterial activity. Phykos.

[23] Anwer S.S., Abdulkareem P.M. (2014). Antibacterial activity of *Lyngbya* and *Chroococcus* species isolated from koya (Hizoop River). J. Life Sci..

[24] Prakash J.W., Marimuthu J., Jeeva S. (2011). Antimicrobial activity of certain fresh water microalgae from Thamirabarani River, Tamil Nadu, South India. Asian Pacific J. Trop. Biomed..

[25] Ostensvik O., Skulberg O.M., Underdal B., Hormazabal V. (1998). Antibacterial properties of extracts from selected planktonic freshwater Cyanobacteria–a comparative study of bacterial bioassays. J. Appl. Microbiol..

[26] Silva-Stenico M.E., Silva C.S.P., Lorenzi A.S., Shishido T.K., Etchegaray A., Lira S.P., Moraes L.A.B., Fiore M.F. (2011). Non-ribosomal peptides produced by Brazilian cyanobacterial isolates with antimicrobial activity. Microbiol. Res..

[27] Ibraheem I.B.M., Al-Othman M.R., Abdelraouf N. (2012). Cyanobacterial extra-metabolites against some pathogenic bacteria. Afr. J. Microbiol. Res..

[28] Gnanamani A., Hariharan P., Paul-Satyaseela M. (2017). Staphylococcus aureus: Overview of bacteriology, clinical diseases, epidemiology, antibiotic resistance and therapeutic approach. Front. Staphylococcus aureus.

[29] Hiramatsu K., Cui L., Kuroda M., Ito T. (2001). The emergence and evolution of methicillin-resistant Staphylococcus aureus. Trends Microbiol..

[30] Karu T. (1999). Primary and secondary mechanisms of action of visible to near-irradiation on cells. J. Photochem. Photobiol. B Biol..

[31] Oron U., Yaakobi T., Oron A., Mordechovitz D., Shofti R., Hayam G., Dror U., Gepstein L., Wolf T., Haudenschild C. (2001). Low-energy laser irradiation reduces formation of scar tissue after myocardial infarction in rats and dogs. Circulation.

[32] Sommer A.P., Pinheiro A.L.B., Mester A.R., Franke R.-P., Whelan H.T. (2001). Bio stimulatory windows in low-intensity laser activation: Lasers, scanners, and nasa’s light-emitting diode array system. J. Clin. Laser Med. Surg..

[33] Hu W.-P., Wang J.-J., Yu C.-L., Lan C.-C.E., Chen G.-S., Yu H.-S. (2007). Helium–neon laser irradiation stimulates cell proliferation through photo stimulatory effects in mitochondria. J. Investig. Dermatol..

[34] Najeeb S., Khurshid Z., Zafar M.S., Ajlal S. (2016). Applications of light amplification by stimulated emission of radiation (lasers) for restorative dentistry. Med. Princ. Pract..

[35] Belykh E., Yagmurlu K., Martirosyan N.L., Lei T., Izadyyazdanabadi H.M., Malik K.M., Byvaltsev V.A., Nakaji P., Preul M.C. (2017). Laser application in neurosurgery. Surg. Neurol. Int..

[36] Allen M.M. (1968). Simple conditions for growth of unicellular blue-green algae on plates 1, 2. J. Phycol..

[37] Allen M.M., Stanier R.Y. (1968). Selective isolation of blue-green algae from water and soil. Microbiology.

[38] Rippka R., Deruelles J., Waterbury J.B., Herdman M., Stanier R.Y. (1979). Generic assignments, strain histories and properties of pure cultures of Cyanobacteria. Microbiology.

[39] Rippka R. (1988). [1] isolation and purification of *Cyanobacteria*. Methods Enzymol..

[40] Cribier B., Prevost G., Couppie P., Finck-Barbancon V., Grosshans E., Piemont Y. (1992). Staphylococcus aureus leukocidin: A new virulence factor in cutaneous infections?. Dermatology.

[41] Rutala W.A., Katz E.B.S., Sherertz R.J., Sarubbi F.A. (1983). Environmental study of a *methicillin-resistant* Staphylococcus aureus epidemic in a burn unit. J. Clin. Microbiol..

[42] Aldayel M., El Semary N. (2020). UV irradiation-promoting effect on the antibacterial activity of cyanobacterial extracts against plant pathogens: A first record. Egypt. J. Biol. Pest Control.

[43] Claus D. (1992). A standardized gram staining procedure. World J. Microbiol. Biotechnol..

[44] Bauer A.W. (1966). Antibiotic susceptibility testing by a standardized single disc method. Am. J. Clin. Pathol..

[45] El Semary N., Al Dayel M., Al Amer K., Al Ali K.M. (2020). Investigating the applications of Chlorella vulgaris in agriculture and nanosilver production. J. Environ. Biol..

[46] Heidari F., Riahi H., Yousefzadi M., Asadi M. (2012). Antimicrobial activity of Cyanobacteria isolated from hot spring of geno. Middle-East J. Sci. Res..

[47] Sherwood A.R., Presting G.G. (2007). Universal primers amplify a 23S rDNA plastid marker in eukaryotic algae and Cyanobacteria 1. J. Phycol..

[48] Dellaporta S.L., Wood J., Hicks J.B. (1983). A plant DNA minipreparation: Version II. Plant Mol. Biol. Report..

[49] Presting G.G. (2006). Identification of conserved regions in the plastid genome: Implications for DNA barcoding and biological function. Botany.

[50] Hajnalka H., Kovács A.W., Riddick C., Présing M. (2013). Extraction methods for phycocyanin determination in freshwater filamentous Cyanobacteria and their application in a shallow lake. Eur. J. Phycol..

[51] Zavřel T., Chmelík D., Sinetova M.A., Červený J. (2018). Spectrophotometric determination of phycobiliprotein content in cyanobacterium synechocystis. J. Vis. Exp..

[52] Tan H.T., Khong N.M.H., Khaw Y.S., Ahmad S.A., Yusof F.M. (2020). Optimization of the freezing-thawing method for extracting phycobiliproteins from *Arthrospira* sp.. Molecules.

[53] Jerez-Martel I., García-Poza S., Rodríguez-Martel G., Rico M., Afonso-Olivares C., Gómez-Pinchetti J.L. (2017). Phenolic profile and antioxidant activity of crude extracts from microalgae and Cyanobacteria strains. J. Food Qual..

[54] Sikkema J., de Bont J.A., Poolman B. (1995). Mechanisms of membrane toxicity of hydrocarbons. Microbiol. Rev..

[55] Fastner J., Heinze R., Humpage A.R., Mischke U., Eaglesham G.K., Chorus I. (2003). Cylindrospermopsin occurrence in two german lakes and preliminary assessment of toxicity and toxin production of *Cylindrospermopsis raciborskii* Cyanobacteria isolates. Toxicon.

[56] Monserrat J.M., Yunes J.S., Bianchini A. (2001). Effects of *Anabaena spiroides* (Cyanobacteria) aqueous extracts on the acetylcholinesterase activity of aquatic species. Environ. Toxicol. Chem. Int. J..

[57] El Semary N.A., Osman M., Botros H., Sabry A., Farag A.T. (2015). Antibiosis towards human and plant pathogens via radiation-induced *Synechococcus elongates* nageli. Egypt. J. Biol. Pest Control.

[58] Xue L., Zhang Y., Zhang T., An L., Wang X. (2005). Effects of enhanced ultraviolet-B radiation on algae and Cyanobacteria. Crit. Rev. Microbiol..

[59] Castenholz R.W., Garcia-Pichel F. (2012). Cyanobacterial responses to UV radiation. Ecology of Cyanobacteria II.

[60] Ahmed E.S., Keyba H.M., Moussa L.A. (2014). Gamma ray induced effects on new species of Streptomyces (aefo2) (hm775973. 1gi: 302495616) isolated from egyptian soil. Int. J. Bioassays.

[61] Fatima N., Ahmad I.Z., Chaudhry H. (2017). Alterations in the antibacterial potential of *Synechococcus* spp. pcc7942 under the influence of UV-B radiations on skin pathogens. Saudi J. Biol. Sci..

[62] Doman K.M., El-Monem M.A., Gharieb M.M. (2020). Effect of ultraviolet-B radiation on the biochemical composition and antibacterial activity of *Spirulina platensis*. J. Basic Environ. Sci..

[63] Kim N.N., Shin H.S., Park H.G., Lee J., Gyung-Suk K., Choi C.Y. (2014). Profiles of photosynthetic pigment accumulation and expression of photosynthesis related genes in the marine Cyanobacteria *Synechococcus* sp.: Effects of led wavelengths. Biotechnol. Bioprocess Eng..

[64] Wen-Tyng L., Yao-Chu L., Jia-Lung W. (2010). Red-light light-emitting diode irradiation increases the proliferation and osteogenic differentiation of rat bone marrow mesenchymal stem cells. Photomed. Laser Surg..

[65] Hsiao-Wei C., Tsung-Shi Y., Mao-Jing C., Yu-Ching C., Eugene I., Chen W., Chen-Lung H., Ying-Jang L., Chi-Cheng Y., Ju-Ching C. (2014). Purification and immunomodulating activity of C-phycocyanin from *Spirulina platensis* cultured using power plant flue gas. Process Biochem..

[66] Sivasankari S., Vinoth M., Ravindran D., Baskar K., Alqarawi A.A., Abd-Allah E.F. (2021). Efficacy of red light for enhanced cell disruption and fluorescence intensity of phycocyanin. Bioprocess Biosyst. Eng..

[67] Wiench R., Skaba D., Stefanik N., Kępa M., Gilowski Ł., Cieślar G., Kawczyk-Krupka A. (2019). Assessment of sensitivity of selected *Candida* strains on antimicrobial photodynamic therapy using diode laser 635 nm and toluidine blue–in vitro research. Photodiagnosis Photodyn. Ther..

[68] Bjordal J.M., Couppé C., Chow R.T., Tunér J., Ljunggren E.A. (2003). A systematic review of low level laser therapy with location-specific doses for pain from chronic joint disorders. Aust. J. Physiother..

[69] Tang J., Li L., Li M., Du L., Waleron M., Waleron M., Waleron K., Daroch M. (2021). Description, taxonomy, comparative genomics of a novel *Thermoleptolyngbya* strain isolated from hot springs of Ganzi, Sichuan China. bioRxiv.

[70] Ki-Seok Y., Nguyen N.T., Tran K.T., Tsuji K., Ogo S. (2017). Nitrogen fixation genes and nitrogenase activity of the non-heterocystous cyanobacterium *Thermoleptolyngbya* sp. o-77. Microbes Environ..

[71] Tang J., Jiang D., Luo Y., Liang Y., Li L., Shah M.M.R., Daroch M. (2018). Potential new genera of cyanobacterial strains isolated from thermal springs of western Sichuan, China. Algal Res..

[72] Sciuto K., Moro I. (2016). Detection of the new cosmopolitan genus *Thermoleptolyngbya* (Cyanobacteria, leptolyngbyaceae) using the 16S rRNA gene and 16S–23S its region. Mol. Phylogenetics Evol..

[73] Momper L., Hu E., Moore K.R., Skoog E.J., Tyler M., Evans A.J., Bosak T. (2019). Metabolic versatility in a modern lineage of Cyanobacteria from terrestrial hot springs. Free Radic. Biol. Med..

